# A Community Mental Health Integrated Disaster Preparedness Intervention for Bushfire Recovery in Rural Australian Communities: Protocol for a Multimethods Feasibility and Acceptability Pilot Study

**DOI:** 10.2196/53454

**Published:** 2024-06-04

**Authors:** Caitlin E Pike, Henriette C Dohnt, Phillip J Tully, Warren Bartik, Courtney Welton-Mitchell, Clara V Murray, Kylie Rice, Suzanne M Cosh, Amy D Lykins

**Affiliations:** 1 School of Psychology University of New England Armidale Australia; 2 Colorado School of Public Health University of Colorado Colorado, CO United States

**Keywords:** bushfires, wildfires, rural mental health, natural disasters, mental health, disaster preparedness, natural hazards resilience, community interventions, mixed-methods, pilot study, disaster, preparedness, preparation, natural hazard, psychological distress, resilience, help-seeking

## Abstract

**Background:**

Natural hazards are increasing in frequency and intensity due to climate change. Many of these natural disasters cannot be prevented; what may be reduced is the extent of the risk and negative impact on people and property. Research indicates that the 2019-2020 bushfires in Australia (also known as the “Black Summer Bushfires”) resulted in significant psychological distress among Australians both directly and indirectly exposed to the fires. Previous intervention research suggests that communities impacted by natural hazards (eg, earthquakes, hurricanes, and floods) can benefit from interventions that integrate mental health and social support components within disaster preparedness frameworks. Research suggests that disaster-affected communities often prefer the support of community leaders, local services, and preexisting relationships over external supports, highlighting that community-based interventions, where knowledge stays within the local community, are highly beneficial. The Community-Based Disaster Mental Health Intervention (CBDMHI) is an evidence-based approach that aims to increase disaster preparedness, resilience, social cohesion, and social support (disaster-related help-seeking), and decrease mental health symptoms, such as depression and anxiety.

**Objective:**

This research aims to gain insight into rural Australian’s recovery needs post natural hazards, and to enhance community resilience in advance of future fires. Specifically, this research aims to adapt the CBDMHI for the rural Australian context and for bushfires and second, to assess the acceptability and feasibility of the adapted CBDMHI in a rural Australian community.

**Methods:**

Phase 1 consists of qualitative interviews (individual or dyads) with members of the target bushfire-affected rural community. Analysis of these data will include identifying themes related to disaster preparedness, social cohesion, and mental health, which will inform the adaptation. An initial consultation phase is a key component of the adaptation process and, therefore, phase 2 will involve additional discussion with key stakeholders and members of the community to further guide adaptation of the CBDMHI to specific community needs, building on phase 1 inputs. Phase 3 includes identifying and training local community leaders in the adapted intervention. Following this, leaders will co-deliver the intervention. The acceptability and feasibility of the adapted CBDMHI within the community will be evaluated by questionnaires and semistructured interviews. Effectiveness will be evaluated by quantifying psychological distress, resilience, community cohesion, psychological preparedness, and help-seeking intentions.

**Results:**

This study has received institutional review board approval and commenced phase 1 recruitment in October 2022.

**Conclusions:**

The study will identify if the adapted CBDMHI is viable and acceptable within a village in the Northern Tablelands of New South Wales, Australia. These findings will inform future scale-up in the broader rural Australian context. If this intervention is well received, the CBDMHI may be valuable for future disaster recovery and preparedness efforts in rural Australia. These findings may inform future scale-up in the broader rural Australian context.

**International Registered Report Identifier (IRRID):**

DERR1-10.2196/53454

## Introduction

### Background

Due to climate change, natural hazards are increasing in frequency and intensity in Australia [1-4]. Natural hazards are defined as “threatening events, capable of producing damage to the physical and social space where they take place;” a natural hazard becomes a natural disaster when it has “a major impact on society and/or infrastructure” [5]. The 2019-2020 bushfires followed 3 years of intense drought in southern and eastern Australia and lasted nearly 12 months in some parts of the country. The bushfires burned an estimated 60 million acres, destroyed 6000 buildings (mostly homes), were responsible for the deaths of 462 people (33 fire-related and 426 from smoke inhalation) and contributed to the deaths of an estimated 3 billion animals [6-10]. The loss of land, displacement of people, and related myriad direct and indirect impacts of the 2019-2020 bushfires on millions of Australians were unprecedented [1]. Most communities were ill-equipped to cope with the scale of the fires.

The 2019-2020 bushfires resulted in significant psychological distress for both directly and indirectly exposed Australians [11]. Direct exposure to bushfires includes but is not limited to (1) loss of property, infrastructure, livelihood, and life; (2) impact of smoke inhalation on both physical and mental health; and (3) first-hand exposure to bushfire events. The negative impact of direct exposure on psychological well-being is extensively documented [12]. Indirect exposure to bushfires can occur through watching, listening, or reading news reports; witnessing someone being negatively impacted by bushfires; exposure to bushfire content through social media (eg, Instagram and Facebook); and discussions within the community about bushfire events. The impact of indirect exposure on psychological well-being mimics the experiences of those who are directly exposed [13]. Exposure to natural disasters in general can have long-lasting psychological consequences for individuals and their communities. On an individual level, a third to half of the exposed population can experience transient distress following the catastrophic event and a significant proportion may go on to develop chronic psychological conditions such as posttraumatic stress disorder (PTSD), depression, anxiety disorders, substance use, and suicidality [14-17]. Bushfire-specific research on the Ash Wednesday, Black Saturday, and Black Summer bushfires shows that these disorders and elevated psychological distress can persist for years or even decades, with distress directly proportional to bushfire proximity and severity [18-20]. These psychological consequences can be compounded upon repeated exposure to additional traumatic events or stressful life events [21-23].

The more physically prepared individuals are for natural disasters, the less physical and psychological harm they are likely to experience [24]. Yet, evidence suggests that communities and individuals often do not engage in disaster preparedness, even when sufficient education and resources are provided, and even in areas with past hazard exposure [24-27]. It has been found that individuals overestimate their level of preparedness. For example, a US study using national survey data found that 78% of respondents reported being prepared for disasters, whereas only 45% were actually prepared by objective assessment [28]. Preexisting mental health concerns and mental health consequences of prior disaster exposure may also interfere with disaster preparedness [29,30]. Research has shown that community members with poor mental health are less likely to have either an emergency plan or household disaster supplies [29]. Possible explanations for unpreparedness include cognitive and behavioral avoidances, commonly found in PTSD, and a sense of hopelessness, commonly found in depression, as well as procrastination and denial [30].

It is important to address barriers to disaster preparedness and enhance community resilience. Resilience in relation to natural disasters encompasses 3 common themes, first, the ability to absorb or accommodate the stressor or adverse event; second, the ability to recover and return to healthy levels of functioning (and not necessarily predisaster functioning); third, the ability to learn, adapt, or transform in response to disaster exposure [1,31-35]. The Australian Disaster Resilience Index was created to assess disaster resilience in Australia and it was found that most of the population of Australia lived in areas that were assessed as having “moderate” resilience for natural disasters, with “low” disaster resilience observed primarily in remote areas [1]. Most of the Australian population has a “moderate” ability to prepare for, absorb, and recover from natural disasters, and learn to adapt and problem solve. Natural hazards resulting in disasters have the potential to adversely affect the social cohesion of communities, which in turn, is linked with disaster resilience [31]. For example, disasters can rupture social networks within communities, thereby eroding existing social relationships and undermining social support [14,36]. Research also indicates that “cognitive social capital” matters, specifically, that lower levels of trust and a diminished sense of belonging are associated with poor mental health in disaster-affected communities [37]. Reciprocal relationships are important as limited social connections following disaster exposure have been associated with an increased risk of PTSD [38]. Taken together, this body of research underscores the importance of social support and community trust as protective factors associated with mental health in disaster-affected settings. Such factors can enhance or diminish community resilience.

Community-based initiatives that encourage collective involvement in mitigation, preparedness, response, and recovery can build resilience to natural hazards. It is preferable for local community leaders to collaborate with community members in developing local preparedness plans and related initiatives, collectively investing in resilience building [25]. Perhaps not surprisingly, studies suggest that disaster-affected communities often prefer the support of community leaders over that provided by health practitioners or other outsiders [39]. Collective community action associated with preparedness may also help to facilitate social cohesion, in turn having a positive impact on individual and collective well-being [40]. In summary, mental health, social cohesion, and disaster preparedness are important factors to address simultaneously through community-based programs to enhance resilience linked to disaster mitigation, preparedness, response, and recovery [30,40,41].

### Community-Based Disaster Mental Health Intervention

The Community-based Disaster Mental Health Intervention (CBDMHI) is an evidence-based approach to building community resilience in the face of natural hazards-related disasters. It has been implemented and evaluated with earthquake, hurricane, and flood-affected communities in Haiti and Nepal [25,30,42-44]. In addition to this initiative, it is in the process of being adapted and further tested in India, Turkey, and Syria for disaster-affected populations. The CBDMHI intervention aims to increase disaster preparedness, resilience, community cohesion, and social support (disaster-related help-seeking) and decrease mental health symptoms, such as depression, anxiety, and PTSD. A 3-day manualized version of the CBDMHI intervention is available in English, Haitian Kreyol, and Nepali. Previous research across 3 studies—2 randomized controlled trials and a matched cluster comparison—with disaster-affected communities in Haiti and Nepal support its effectiveness in increasing preparedness and social cohesion and decreasing mental health symptoms [25,30]. The intervention is specifically designed to be adaptable across contexts, cultures, and specific events. Community members are part of this adaptation process and are trained to cofacilitate the intervention. An initial consultation phase is a key component of this adaptation process.

### Study Objectives

This project aims to adapt the CBDMHI for the Australian bushfire context to increase community resilience in the face of ongoing bushfire risk. After adapting to the local context and for fires, the aim is to test the adapted intervention in a small-scale pilot study to assess the feasibility, acceptability, and effectiveness of the intervention in 1 rural community to inform potential scale-up for other disaster-affected communities in Australia.

## Methods

### Study Design

**Figure 1:**
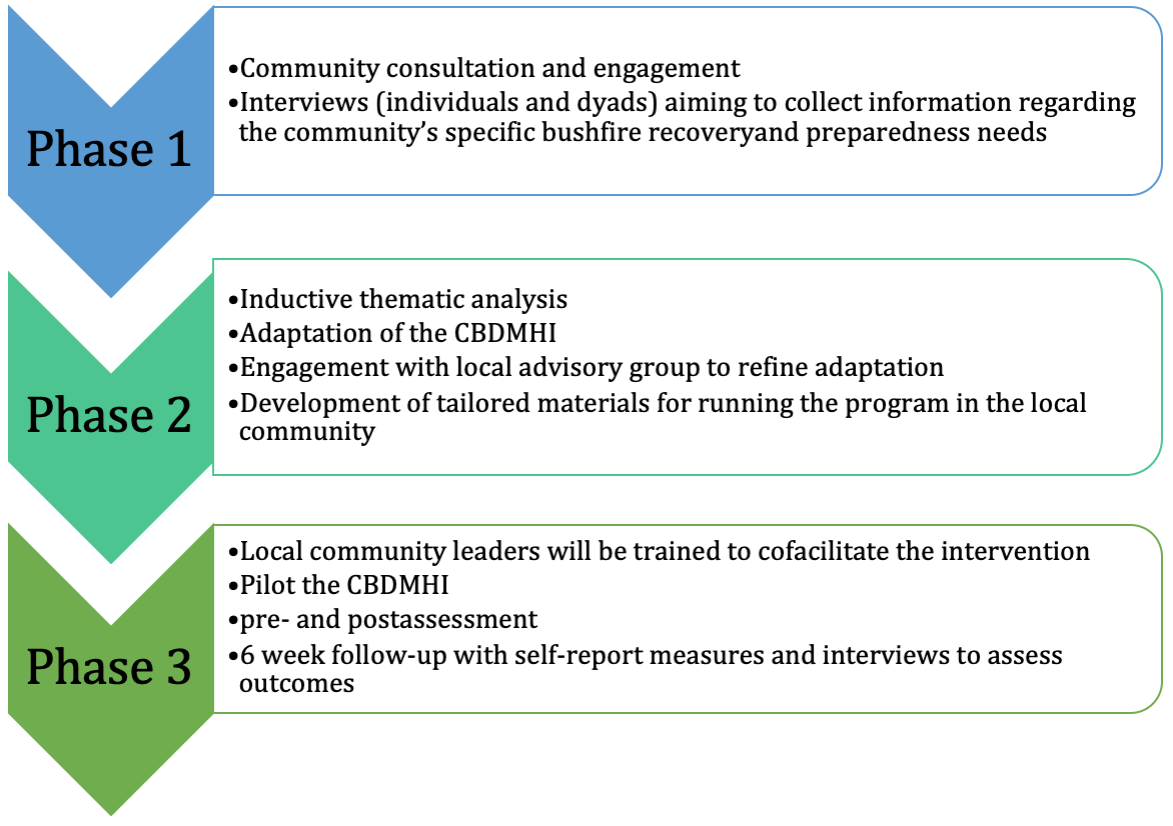
Study phases. CBDMHI: Community-Based Disaster Mental Health Intervention.

This research uses a sequential multimethods approach. This research is broken into 3 phases that are outlined in Figure 1. Phase 1 assesses the needs of the community to appropriately adapt the CBDMHI. Phase 1 consists of qualitative interviews (individual or dyads) with self-identifying members of a rural community in New South Wales, Australia, aiming to collect information regarding the community’s specific bushfire recovery needs. This process will include identifying themes related to disaster preparedness, social cohesion, and mental health. Phase 2 will involve additional discussion with key members of the community to refine the adaptation of the CBDMHI to specific community needs, building on phase I inputs. Local community leaders will be trained to cofacilitate the intervention in the community, which will be piloted in phase 3 of this study. Intervention acceptability, feasibility, and preliminary effectiveness will be assessed via pre- and postintervention individual mental health and community variables, as well as a 6-week follow-up. Acceptability will also be assessed postintervention.

Phase 1 will focus on community consultation and engagement. Community awareness events will be undertaken in the identified community prior to the commencement of the project, to promote the project and obtain community support (eg, phone calls with individuals in the community, face-to-face meetings in the local café to promote the project, and events with the local fire service). Phase 1 will primarily involve data collection through semistructured interviews with open-ended questions. The interviews will explore the impact of the 2019-2020 bushfires on community cohesion, community resilience, and well-being, as well as intervention preferences and perceived needs. Interviews will also focus on community preparedness for the 2019-2020 bushfires and what measures have been taken since that time to prepare for future fires. Questions will focus on current perceived needs related to the fires (at both the individual and community levels), primary stressors (past and current), and ongoing recovery. The data gathered will be used to inform the adaption of the CBDMHI to the local bushfire context.

In phase 2, the CBDMHI will be adapted to meet specific community needs. Data from phase 1 will be analyzed using inductive thematic analysis at the semantic level. The findings of the qualitative analysis will inform the adaptation of the CBDMHI. Adaptation based on community feedback may include the scenarios and group activities so that they are fire and community-specific; the content covered or focused on in the program (ie, the content will be selected to match stated community needs, for example, based on community feedback, there may be more of a focus on household level preparedness than on assisting extremely vulnerable groups within the community); and the length and timing of the program. The adaptation process may also necessitate additional content being added by clinical experts through the inclusion of additional psychoeducation or skills training to meet the community’s needs. Phase 2 will also involve additional discussion with key members of the community to further refine the adaptation of the CBDMHI to specific community needs. A local advisory group of community leaders and members will be formed for consultation during the adaptation process. Tailored materials for running the program in the local community will be developed.

Phase 3 will involve piloting the adapted CBDMHI in the identified rural community. Phase 3 has been registered with the Australian New Zealand Clinical Trials Registry (ANZCTR) registration (ACTRN12623001023640). As part of this phase, the research team will train local and respected community leaders to administer or co-deliver the adapted CBDMHI. The intervention will consist of facilitated discussion, the space to share personal experiences, the establishment of peer support, the establishment of safety, and learning and practicing coping skills specifically for disaster-related distress. The intervention also provides practical training in disaster preparedness and response techniques that participants can use to support themselves and other community members during future hazards.

Phase 3 of the study will pilot the CBDMHI with residents of the affected community. The pilot intervention will use a pretest and posttest design to assess the acceptability of the intervention, as well as levels of psychological distress, resilience, coping, community cohesion, psychological preparedness, and help-seeking intentions following the intervention. Participants will complete measures before and after the workshop to assess the treatment effectiveness, as well as a 6-week follow-up. Due to the community setting and different availabilities of community members living in rural areas with property and farming obligations, random allocation is unlikely to be possible. Workshop participation and thus treatment implementation will, therefore, be a within-subject design with participants acting as their own controls. Drawing on the results from this study, a framework with Australian bushfire-specific materials will be developed and made available to facilitate broader implementation in the future to support recovery in other fire-affected Australian communities.

### Eligibility Criteria

Eligible participants are (1) persons aged 18 years and older, (2) who self-identify as a member of the bushfire-affected community in the Northern Tablelands region of New South Wales, (3) have lived and worked in the community during the fires and continue to reside within the bushfire-affected community, and (4) report having been directly or indirectly impacted by the 2019-2020 bushfires. Direct exposure includes having protected property or livestock; having been evacuated or relocated; experiencing loss of property, family, friends, and livestock; or having served as a firefighter or first responder, including in a volunteer capacity. Indirect exposure includes having heard recounts of the bushfires and their effects on the community secondhand. Ineligible participants are those not fluent in English or individuals who reside outside of the recruitment catchment area or are judged by clinicians as under the influence of alcohol or substances or have any other significant impediment to participation.

### Proposed Sample

The proposed sample for phase 1 will be determined through informational power [38]. Informational power is based on 5 concepts, aim, specificity, theory, dialogue, and analysis. When informational power is met the sample size will be determined.

The proposed sample for phase 3 is up to 20 participants, which is estimated as more than 15% of the total population of the target community. This target sample size is based on previous adaptations of the CBDMHI that used no more than 20 participants per intervention group [30,42]. This number is also in line with the recommended treatment group size for pilot studies [45].

### Recruitment

#### Phases 1 and 2

Participants will be recruited via advertising within the affected community, including community services, dining venues, and schools. The project will be advertised through partner organizations including the Rural Fire Service (RFS), community groups, and local social media pages, and through community engagement events held with the RFS.

Advertising materials and social media posts will be shareable, permitting recruitment through social contagion and snowball techniques, allowing maximum reach and community representation. The project team will contact individuals who registered their interest by phone and email to confirm eligibility and schedule interviews. Participation will not be incentivized.

#### Phase 3

This phase is open to any of the phase 1 participants, as well as any other persons residing or working in the catchment area (as per the eligibility criteria). Recruitment will occur through advertising within the target community including through the mentioned partner organizations, community services, community groups, and social media pages, and during local community events.

### Intervention

The CBDMHI is a manualized intervention that is culturally adaptable to disaster-affected communities [42]. Prior to the intervention, the research team will select and train local community leaders to deliver the modified CBDMHI. The CBDMHI will be modified in content and length based on the local communities’ needs and availability. The main objectives of the CBDMHI are to increase disaster preparedness (physical and psychological), community cohesion, resilience, and social support (disaster-related help-seeking), and decrease mental health symptoms, such as depression, anxiety, and PTSD.

### Outcomes

#### Measures

Participants in phase 3 of this research will complete a range of self-report measures prior to the intervention, at completion of the intervention, as well as a 6-week follow-up to assess durability. Measures will be collected via an anonymous survey completed in person with trained assessors pre- and postintervention and follow-up measures will be emailed along with reply paid envelopes. To link pre- and posttest questionnaire results, participants will be assigned a unique identifier.

#### Acceptability

Acceptability of the intervention will be measured using Likert-style and open-ended questions. The Likert-style questions will be scored using a 5-point Likert scale ranging from “strongly disagree” to “strongly agree,” with higher scores indicating higher acceptability. Open-ended questions (eg, “what did you like about the workshop?”) will also be used to measure acceptability. Participants will also be invited to take part in an optional follow-up semistructured interview to assess acceptability and perceived unhelpfulness.

#### Psychological Distress

Psychological distress will be measured using the Kessler Psychological Distress Scale (K10) [46]. The K10 is a 10-item questionnaire based on questions about anxiety and depressive symptoms that an individual has experienced in the previous 4 weeks. The K10 is based on a 5-point Likert scale, with responses ranging from “none of the time” to “all of the time.” The maximum score is 50 (indicating severe distress, likely to have a severe disorder), and the minimum score is 10 (indicating low distress, likely to be well). The K10 has demonstrated favorable discriminant validity and internal consistency [47].

#### Resilience

Resilience will be assessed through the Brief Resilience Scale (BRS), which aims to assess an individual’s ability to bounce back or recover from stress [48]. The BRS is scored on a 5-point Likert scale ranging from “strongly agree” to “strongly disagree” and consists of 6 items. Item scores are summed and then divided by 6, so that mean scores range from 0 to 6. Mean scores of 1.00 to 2.99 reflect low resilience, 3.00 to 4.30 reflect normal resilience and 4.31 to 6.00 reflect high resilience. The BRS is a reliable measure of assessing resilience [48].

#### Social Cohesion

Social cohesion will be measured using items adapted from Sampson et al [49]. The adapted measure consists of 5 questions asking respondents to rate neighborhood’s perceived closeness of residents, willingness to help, trust, conflict, and values. The 5 items are each scored on a 5-point Likert scale ranging from “strongly agree” to “strongly disagree” [49]. Total scores range from 5 to 25, with higher scores representing a higher sense of community.

#### Help-Seeking

The help-seeking intention will be measured using 2 adapted items from the CBDMHI developers [30] that ask respondents “Would you be comfortable seeking help from others if you were experiencing sadness, stress or burnout?” and “Would you be comfortable seeking help from others if you needed something to prepare for or in the aftermath of a disaster?” Response options are scored on a 4-point scale ranging from “I would not be comfortable at all” to “I would be very comfortable.” Total scores range from 2 to 8, with higher scores representing higher help-seeking intention.

#### Coping

Coping will be measured using an adapted version of the Brief-Coping Orientation to Problems Experienced Inventory (Brief-COPE) [50]. The adapted coping measure consists of 4 questions targeting acceptance, denial, self-distraction, and solution-focused coping. Items are scored on a 5-point Likert scale ranging from “None of the time” to “All of the time.” Higher scores represent higher engagement in a given coping style, and are, therefore, evaluated as individual items.

#### Psychological Preparedness

Psychological preparedness will be measured using the Psychological Preparedness for Disaster Threat Scale (PPDTS) [51]. The PPDTS is a self-report questionnaire that consists of 18 items, each scored on a 4-point Likert scale ranging from “not at all true of me” to “exactly true of me” [51]. Higher scores represent higher perceived psychological preparedness [51].

### Planned Data Analysis

Descriptive statistics will be provided for demographic data. Phase 1 community consultation interview data will be analyzed using thematic analysis [52] to explore participants’ physical, psychological, social, and environmental needs following the 2019-2020 bushfires. Inductive thematic analysis has been selected for this study as it is a useful qualitative approach for applied research designs [53]. Thematic analysis provides a flexible yet robust, systematic framework for coding data and has been used widely in the health sciences [53]. Data analysis will involve an iterative process including familiarizing oneself with the data, generating initial codes, searching for themes, reviewing themes, defining and naming themes, and producing the report [52,54].

One-way repeated measures ANOVAs will be performed for phase 3 outcome measures to identify any changes in pre- and postintervention outcomes, and whether those changes were maintained up to 6 weeks. Multilevel mixed models nested in time may also be used, as these analyses are more robust to missing data and can be used to model change trajectories over time. Where a change in score across time points is identified, the reliable change index will be used to assess the clinical significance of this change to provide further insight into the clinical meaningfulness and use of the program.

### Ethical Considerations

Phases 1 and 3 of this study were approved by the University of New England Human Research Ethics Committee (HE22-120 and HE23-083, respectively). Written or recorded informed consent will be obtained from the participants before inclusion in either phase of the study. Study data will be deidentified and any potentially identifiable information will be removed from any published materials to ensure privacy. Participants will be reimbursed for their time through meals and hot drinks being offered during interviews, and through opportunities for participants to win vouchers to local businesses (eg, local café and pub). Study participants are free to withdraw at any time without providing a reason.

## Results

Data collection commenced following support from the Black Summer Recovery Grant Scheme. As of January 2023, more than 10% of the identified rural community in the Northern Tablelands has participated in phase 1 interviews. These interviews have been transcribed and a thematic analysis of the data is currently taking place. As of September 2023, a total of 10 rural community members participated in the phase 3 intervention. Follow-up data were collected in November 2023, and the first results are to be submitted for publication in 2024.

## Discussion

### Overview

The predicted rise in frequency and intensity of extreme weather events underscores the importance of disaster preparation and resilience. Australia has been assessed as currently having a “moderate” level of resilience, with “low” resilience in many rural areas [1], underscoring the critical need for resilience interventions in Australia. Community involvement in recovery positively impacts community resilience, and resilient communities are less vulnerable to the negative effects of natural hazards [32]. The CBDMHI is valuable as it is community-focused and aims to keep the knowledge and skills within the local community. The development of the CBDMHI means that community needs have to be ascertained before the intervention can be adapted, adding to the growing research and literature surrounding natural hazards, preparedness, and mental health in rural Australia. By using community consultation and an advisory group of community members in phases 1 and 3, it is anticipated that acceptability will be high. The local advisory group can also aid in the feasibility of the intervention by ensuring that its design facilitates the participation of community members and reduces barriers to access.

### Comparison With Prior Work

Previous research has found that the adapted CBDMHI resulted in increased disaster preparedness (both physical and psychological), community cohesion, social support (disaster-related help-seeking), and resilience and a decrease in psychological distress. Targeting both preparedness and mental health has direct effects on individual and community variables that are also mediated by both mental health training and preparedness training [42]. The CBDMHI will include coping skills and preparedness training to alleviate psychological distress associated with bushfires and to try to increase preparedness for future bushfires. It is hoped that the participants of this pilot study will experience similar benefits to previous communities who have used the CBDMHI. It is hoped that the adapted intervention will decrease psychological distress and increase resilience, coping, community cohesion, psychological preparedness, and help-seeking intentions following the intervention.

Adaptations to the CBDMHI are likely to include bushfire-specific preparedness techniques and be more specific to the Australian context and the cultural makeup of rural communities. If the adapted CBDMHI is feasible and acceptable in this rural Australian community, it will provide a framework for other adaptations to disaster- and fire-affected communities within Australia. For instance, the adapted CBDMHI could be tested and evaluated with other communities affected by bushfires. It could also be further adapted and tested with communities affected by major flood events, such as the Northern Rivers region of New South Wales in Australia, which experienced several major floods within the same calendar year (2022) [55].

### Limitations

While improvements in target outcomes are likely to increase the community’s resilience and preparedness for future disasters, this study is proposed with several limitations. First, the sample size is small and potentially only viable for a pretest and posttest design. In the context of the identified rural Australian community, the intervention may reach 10%-50% of the region’s population, with the small village size and sparse population limiting the feasibility of controlled trials or multiple baseline designs. Further, the intervention findings may not generalize to other regions or other natural hazard events. Even if the adapted CBDMHI is effective and feasible, the findings will require replication with other rural communities and those exposed to other hazards. Second, the length of the follow-up on this study is 6 weeks. While this is appropriate to gain insight into whether the effects of the intervention are maintained over time, the research would be strengthened by having longer follow-up times to better assess the durability of the intervention. Finally, document review was not used as complementary data collection. Having document review alongside the interview and quantitative analysis may have provided more insight into resilience and preparedness in Australian communities helped with cross-verification of community experiences and mitigated against selection bias. Future research could consider this option for data collection at study commencement.

### Conclusions

This protocol describes a multimethods pilot feasibility study focused on adaptation of a previously successful postdisaster intervention, which has been shown to effectively increase disaster preparedness, reduce psychological distress, and improve social cohesion in affected communities in Haiti and Nepal [25,30]. To date, psychological interventions in the Australian bushfire context have focused on providing low-level psychological support to reduce and prevent the development of psychological disorders (eg, Skills for Life Adjustment and Resilience [SOLAR]) and have been piloted with a small sample in the Victorian community [56]. The proposed study differs in that it will integrate mental health in a standard disaster preparedness training, work in partnership with the RFS, use locally based community trainers, and be delivered in a community-based setting. These differences may allow for a realistically scalable intervention for rural communities that are often the most negatively affected by particularly severe bushfire seasons.

## Abbreviations

ANZCTR: Australian New Zealand Clinical Trials Registry
Brief-COPE: Brief-Coping Orientation to Problems Experienced Inventory
BRS: Brief Resilience Scale
CBDMHI: Community-Based Disaster Mental Health Intervention
K10: Kessler Psychological Distress Scale
PPDTS: Psychological Preparedness for Disaster Threat Scale
PTSD: post-traumatic stress disorder
RFS: Rural Fire Service
SOLAR: Skills for Life Adjustment and Resilience

## Data Availability

The data sets generated and analyzed during this study are available from the corresponding author on reasonable request.
